# Differences in Psychological Coping with Illness During the Treatment and Survivorship Phases in Adolescents According to Age and Sex

**DOI:** 10.3390/healthcare12232476

**Published:** 2024-12-06

**Authors:** Diego-José Sáez-Rodríguez, Juan-Manuel Ortigosa-Quiles, Antonio Riquelme-Marin, Raquel Suriá-Martínez, Pablo Chico-Sánchez

**Affiliations:** 1 Department of Personality, Assessment and Psychological Treatments, Faculty of Psychology, University of Murcia, Espinardo University Campus, 30100 Murcia, Spain; ortigosa@um.es (J.-M.O.-Q.); riquelme@um.es (A.R.-M.); 2 Department of Communication and Social Psychology, Social Sciences Building, University Institute for Gender Studies Research, University of Alicante, 03690 Alicante, Spain; raquel.suria@ua.es; 3 Epidemiology Unit, Preventive Medicine Service, Dr. Balmis General University Hospital, Alicante Institute for Health and Biomedical Research (ISABIAL), 03010 Alicante, Spain; chico_pab@gva.es; 4 Department of Community Nursing, Preventive Medicine and Public Health and History of Science, University of Alicante, 03690 Alicante, Spain

**Keywords:** personal resources, cancer, coping, illness period, adolescence

## Abstract

Introduction: Despite the importance of addressing different stages of cancer, there is a lack of data on how these stages relate to coping strategies. This study aims to analyze coping strategies among adolescents with cancer by comparing two distinct time points, the treatment phase and the post-treatment phase, with a particular focus on age and gender. Methodology: A total of 201 cancer patients aged 12 to 17 years from Alicante, Valencia, and Madrid participated in the study. They completed a Demographic and Clinical Data Form questionnaire capturing age, gender, and illness phase, along with the ACS to assess coping strategies. Results: During the treatment phase, strategies such as “worrying”, “stress reduction”, “ignoring the problem”, and “self-blame” were used more frequently than during the follow-up phase, revealing notable changes in emotional management between the two stages. Gender differences were observed in the first three strategies. Conclusions: The coping strategies of adolescents with cancer vary significantly between the treatment and follow-up phases, with greater use of certain strategies during treatment and a decline in their use post-treatment. These findings highlight the evolving emotional demands of each stage and emphasize the need for targeted interventions that address the specific coping needs unique to each phase. Such targeted interventions in clinical settings could support emotional management by adapting strategies to the distinct challenges faced by adolescents during treatment and post-treatment phases.

## 1. Introduction

Oncologic diseases, regarded as chronic disorders with multifactorial causes, affect individuals of all ages, including children and adolescents, with both known and unknown causes [1]. Understanding the physical, psychological, and social factors influencing these patients, particularly in the pediatric and adolescent groups, is crucial [2]. The challenge of low early detection in these groups [3] and the high demand for resources [4] complicates prevention efforts, making it a significant issue [5]. In Spain, 951 children aged 0–14 years were diagnosed with cancer in 2022, with a mortality rate of 2 per 100,000 population [6].

Cancer in adolescents is associated with an increased risk of psychological difficulties, including depression and anxiety, particularly among those facing prolonged physical restrictions [7]. This age group may experience heightened vulnerability, panic, and isolation, which deeply impacts both them and their families [8]. While the challenges of cancer are significant, adolescents also view the disease as an opportunity for transformation and personal growth, accepting negative thoughts such as fear of death and uncertainty [9].

The effects of cancer on adolescents are still not well understood [10]. A life-threatening illness during this developmental stage can lead to more severe psychosocial consequences compared to childhood [11]. Adolescents may experience increased dependence on caregivers, a reduced capacity for social interaction, and difficulties with typical adolescent developmental tasks [12,13]. Moreover, these patients face persistent challenges during treatment, particularly in managing change and uncertainty, which are discussed across five dimensions: diagnosis, interaction with healthcare systems, living with the disease, coping with treatment repercussions, and managing family and social reactions. Adolescents often employ a mix of constructive strategies and defense mechanisms, such as avoidance. From a transactional perspective, coping is viewed as a dynamic process shaped by constant interaction between the individual and their environment. Unlike a static approach, this perspective highlights how adolescents assess the demands of illness and adjust their emotional and behavioral responses based on available resources like social support or personal resilience mechanisms. Considering the disease’s various phases, the transactional approach provides a more comprehensive view of how coping strategies evolve, especially when compared to older, emotionally mature individuals. The complex nature of coping with cancer may also differ based on age and sex, which can impact adolescents’ emotional resilience and their capacity to manage long-term stressors over time [14].

Psychological mechanisms, including cognitive appraisal and emotional regulation, are central in shaping how adolescents perceive and manage their illness. Additionally, timely medical interventions and supportive care play a critical role in alleviating distress and enhancing coping by giving adolescents a sense of control over their treatment. The associations between treatment factors—such as type, duration, and side effects—and coping mechanisms are crucial for understanding how adolescents manage the challenges of their illness. Education about treatment processes and active involvement in decision-making can strengthen coping [15].

While studies like those of Hensel et al. [16] and Erickson and Steiner [17] have primarily focused on the psychological pathologies faced by adolescents with cancer, other research, such as that by Hullmann et al. [18] and Barakat et al. [19], emphasizes adaptive and positive factors, including quality of life, hope, optimism, and various coping strategies. Adolescents, often facing the prospect of relapse, are encouraged to adopt coping strategies that emphasize acceptance and problem-solving, which prove more beneficial in the long run [20]. Despite the negative connotations associated with relapse, acceptance-based strategies have been shown to foster personal growth, increasing psychological resilience and the ability to cope with the disease’s aftermath for both patients and their families [21,22].

Adolescents undergoing cancer treatment develop crucial skills for managing stress, setting goals, and seeking positive outcomes, which improves their long-term psychosocial well-being [23]. Ensuring a return to their emotional and physical state prior to diagnosis is essential for positive outcomes [24]. Regardless of disease stage, adolescents typically employ similar coping strategies, displaying consistent approaches across different phases [25]. Even after treatment, cancer survivors continue to face challenges, but specific coping strategies help them maintain a positive outlook and manage the long-term physical and psychological consequences of the disease [26,27].

Effective coping strategies improve psychological well-being and adaptation to difficult circumstances, fostering positive skills and reducing negative emotional responses, thereby influencing self-esteem and quality of life [28,29]. Adolescents who rely on external resources like family and healthcare professionals, as well as internal resources like faith and resilience, report better outcomes. Strategies such as focusing on the positive, physical recreation, and hard work are linked to increased well-being, while self-blame and isolation are detrimental to overall health [30,31]. Avoidance and denial have been associated with higher psychological distress, lower quality of life, and reduced self-esteem [32,33].

Given the limited research specifically on age- and sex-based differences in coping during different phases of the illness, there is a pressing need to understand how these factors may impact adolescents’ long-term psychological outcomes. Examining these variables throughout the illness trajectory could identify interventions tailored to different ages and genders, ultimately improving adolescents’ adjustment and emotional resilience. Given the relevance of these factors, it is essential to carry out an exhaustive analysis of coping strategies in adolescents facing an oncological process. Likewise, it is necessary to investigate these variables during the different phases of the disease, such as the treatment period, when the patient is hospitalized and receiving medical care, or the post-treatment and follow-up period, which begins after the patient has been discharged from the hospital. The treatment period spans from admission to the hospital until discharge. The distinction between these phases is significant as it influences personal resources and the social context, which impacts the coping strategies employed [34]. Therefore, this study aims to assess how coping strategies evolve throughout the disease process in adolescents, considering the potential influence of age and gender. It is hypothesized that coping strategies will change significantly during the illness, with age and sex being important factors in these changes.

## 2. Methods

### 2.1. Design

This is a quantitative and descriptive study using a cross-sectional observational methodology.

### 2.2. Participants

The sample selection was conducted through the associations Aspanion and Asion. To determine the necessary sample size, considering our study’s characteristics and the planned statistical analyses, we used the statistical software G*Power version 3.1.9.2. Based on this calculation, with a power of 95%, an alpha level of 5%, and assuming a medium effect size (0.3), 174 participants were required [35]. Aspanion facilitated access to patients from the Hospital General Universitario Dr. Balmis and the Hospital Universitario y Politécnico La Fe. Simultaneously, the Asion association granted access to patients from the Hospital Infantil Universitario Niño Jesús. The inclusion criteria for participation in the study were that patients were between the ages of 11 and 18 and had received an oncological diagnosis. Data collection was conducted over a one-year period, with participation from these three hospitals located in the provinces of Alicante, Valencia, and Madrid, Spain. A convenience sampling method was used to recruit participants, selecting from patients currently supported by Aspanion and Asion.

Contact with the families was made through telephone calls, using the information provided by the associations and strictly adhering to legal confidentiality regulations. During these calls, the study’s objectives and the proposed implementation plan were explained in detail to the parents. Once families provided their consent to participate, the corresponding questionnaire was administered.

In total, the study involved 201 participants who were part of various programs offered by the associations, including social, psychological, and neuropsychological support, leisure and recreational activities, and volunteering, among others. These services were accessible both within the hospital setting and through the support provided by these associations to patients and their families.

### 2.3. Instruments

The final version of the structured interview included the following tools:

(a) Demographic and Clinical Data Form: a custom questionnaire was used to collect information on age, gender, and the duration of the illness, distinguishing between the treatment and follow-up phases;
(b) Adolescent Coping Scale (ACS) by Frydenberg and Lewis: this tool, modified for use with the Spanish population, assesses 18 different coping strategies, including “social support”, “solving problems”, “working hard”, “worry”, “friends”, “belonging”, “wishful thinking”, “not coping”, “tension reduction”, “social action”, “ignoring the problem”, “self-blame”, “keeping self”, “spiritual support”, “focusing on the positive”, “professional help”, “relaxing diversions”, and “physical recreation”. The ACS has demonstrated strong psychometric properties, with a Cronbach’s alpha reliability coefficient ranging from 0.80 to 0.93 across different subscales, indicating good internal consistency. The test was customized to allow participants to provide both general responses (regarding how they handle problems overall) and specific responses (focused on how they manage a particular issue that concerns them or that the counselor, tutor, or clinician wishes to address) [36].

### 2.4. Procedure

The research received approval from the Ethics Committee of the University of Murcia (CEI 2870/2020) and, subsequently, was also validated by the Ethics Committees of the Hospital General Universitario Dr. Balmis and the Hospital Universitario y Politécnico La Fe, in cooperation with Aspanion. Additionally, the Ethics Committee of the Hospital Infantil Universitario Niño Jesús, in cooperation with Asion, granted its authorization.

After obtaining these approvals, access to the study sample was facilitated through an authorization form, which was thoroughly completed. This form contained all the necessary details about the research, enabling parents or guardians to grant their consent, thus allowing for direct assessment with the participants.

First, informed consent was obtained from the young participants themselves. Subsequently, the assessment was organized as a series of self-administered questionnaires conducted online. During this process, participants were assisted by the interviewer, ensuring that the assessment was minimally invasive. This approach allowed patients to complete the required information at times when they were in optimal conditions to collaborate with the project.

### 2.5. Data Analysis

First, a statistical analysis was conducted to examine the differences in coping based on the variable period of illness, using absolute and relative frequencies in percentages. A t-test was performed to compare the mean coping strategies across the different phases of the procedure. Additionally, an analysis of covariance (ANCOVA) was conducted, considering age, sex, and period of illness as covariates in relation to the coping strategies. This analysis assessed both the relationship between these factors and coping, as well as the potential significance of any interaction effects between these variables regarding the use of different coping strategies. Data analysis was performed using the IBM^®^ SPSS Statistics v.26.0 software, with a significance level set at *p* < 0.05.

## 3. Results

According to the period of illness, 37 boys (41.1%) and 53 girls (58.9%) were in the treatment phase, while 45 boys (40.5%) and 66 girls (59.5%) were in the discharge or follow-up phase.

As shown in Table 1, the data reveal a significant mean difference in the ‘worrying’ strategy, with a higher mean during the treatment phase compared to the follow-up phase (t = 2.098, *p* = 0.037). Regarding the ‘stress reduction’ strategy, a higher mean was observed during the treatment phase compared to the follow-up phase (t = 1.860, *p* = 0.064). With respect to the ‘ignoring the problem’ strategy, a higher mean was also observed during the treatment phase in contrast to the follow-up phase (t = 2.337, *p* = 0.020). Similarly, the ‘self-blame’ strategy showed higher values during the treatment phase compared to the follow-up phase (t = 2.855, *p* = 0.005).

With respect to the coping strategy ‘worrying,’ a significant difference was found based on gender (F = 16.207, *p* < 0.001) and period of illness (F = 5.693, *p* = 0.018), with a higher mean observed in women compared to men (t = −4.205, *p* < 0.001), as shown in Table 2.

With regard to the ‘stress reduction’ strategy, a significant difference was also found based on gender (F = 4.833, *p* = 0.029), with a higher mean observed in females compared to males (t = −2.342, *p* = 0.020), as shown in Table 3.

In relation to the coping strategy ‘ignoring the problem,’ a significant difference was found based on sex (F = 36.950, *p* < 0.001) and period of illness (F = 6.821, *p* = 0.010), with a higher mean observed in men compared to women (t = 6.144, *p* < 0.001), as shown in Table 4.

Finally, a significant difference was observed between the coping strategy ‘self-blame’ and the period of illness (F = 8.126, *p* = 0.005), as shown in Table 5. This shift may suggest that individuals begin to re-evaluate their circumstances and coping strategies once they are no longer in the immediate treatment phase, highlighting the dynamic nature of coping mechanisms over time. The statistical significance of these findings underscores the need for tailored interventions that address gender-specific coping styles and take into account the context of treatment phases.

## 4. Discussion and Conclusions

The aim of this study was to examine how coping strategies evolve throughout different stages of illness, with particular focus on how these strategies influence adaptation, considering both age and gender. Beyond identifying coping strategies, this study sought to understand their impact on adaptation during the various phases of cancer treatment.

The results partially support the initial hypothesis that coping strategies change significantly as the disease progresses and that these changes depend on gender. Adolescents’ coping strategies were analyzed at different stages of their cancer treatment, offering new insights into coping dynamics during critical periods.

Consistent with the research by Lie et al. [14], significant variations were observed in the use of strategies like “worrying”, “stress reduction”, “ignoring the problem”, and “self-blame”. The increased use of worrying during the active treatment phase reflects the uncertainty and stress tied to medical procedures and their side effects. These findings underscore that managing uncertainty is crucial for adolescents coping with cancer. The effect sizes of these differences, particularly in Table 1, suggest small to medium effect sizes, indicating meaningful differences in adolescents’ coping experiences across phases.

Contrary to Garcia-Garcia [25] findings, which suggested no significant changes in coping strategies across illness phases, this study found that coping strategies significantly varied between the treatment and follow-up periods. The heightened use of “worrying” during treatment indicates an emotional response to the uncertainties surrounding health, prognosis, and side effects. Adolescents may engage in worrying to gain a sense of control over the unpredictable nature of their illness. Moreover, the emotional and physical stress of treatment can exacerbate feelings of vulnerability, prompting adolescents to focus on their concerns as a coping mechanism. The application of stress-coping theory adds a robust framework to the findings, particularly the idea that coping strategies are dynamic and change with perceived stress levels during treatment and follow-up. This theoretical model aligns well with the observed patterns in adolescents’ coping responses, as stress levels may fluctuate based on treatment phase. Further elaboration on how specific strategies align with Lazarus and Folkman’s coping model (problem-focused vs. emotion-focused coping) could enhance the theoretical depth of these results. For example, strategies like worrying and self-blame may be viewed as emotion-focused coping mechanisms when adolescents perceive limited control over the stressors.

The increased use of “ignoring the problem” during treatment suggests an avoidance or denial strategy, possibly as a way to manage the emotional burden associated with intense medical interventions. These findings are consistent with stress-coping theory, which proposes that individuals use different strategies to manage stressors, and that the chosen strategies influence psychological outcomes in both treatment and follow-up phases.

These results align with studies by Turner-Sack et al. [20], Zebrack and Chesler [22], and Belpane et al. [26], which suggest that avoidance-oriented coping strategies are more prevalent during the active treatment phase. Additionally, Rosenberg et al. [23] noted that adolescents develop critical coping skills during the illness phase, which contribute to better long-term psychosocial outcomes. Gibson et al. [24] further emphasized the importance of adolescents striving to return to their pre-illness state, which plays a key role in their psychological adjustment. Adolescent developmental theory could further complement the discussion on coping dynamics, given that teenagers are still in the process of building resilience and adaptive coping skills. Recognizing this developmental context may clarify why avoidance and self-blame are prominent during treatment, whereas more adaptive strategies may emerge during follow-up.

A notable increase in “self-blame” was observed during the treatment period, in contrast to the follow-up phase. This could reflect the internalization of responsibility for the illness, in contrast to findings by Turner-Sack et al. [20] who found more acceptance-based coping strategies in the same phase. This discrepancy indicates a complexity in adolescents’ emotional responses during treatment.

Gender differences were significant in this study. Females were more likely to worry, particularly during treatment, suggesting a greater emotional sensitivity to the uncertainties associated with cancer. Women also employed more “stress reduction” strategies, which highlights a tendency towards active stress management. In contrast, men were more likely to use avoidance strategies, such as “ignoring the problem”, during the more intensive phases of treatment. This suggests that avoidance may provide short-term relief from emotional distress but may hinder the development of adaptive coping strategies for long-term resilience. These sex differences highlight the need for gender-specific interventions to address the emotional needs of adolescents throughout the stages of illness.

The findings support the view that coping strategies differ by illness phase and gender, as suggested by Shin et al. [27], and emphasize the importance of personalized psychological interventions. These should be tailored to the emotional needs of adolescents during the different stages of cancer, acknowledging both phase-related and gender-related coping patterns.

To implement these findings clinically, psychological support should be tailored to the identified strategies and gender differences. Interventions for boys could focus on encouraging emotional expression and reducing avoidance behaviors, while those for girls might emphasize managing anxiety and improving coping skills for dealing with worry. During treatment, immediate psychological support should address heightened emotional responses, while in follow-up, strategies should focus on promoting resilience and reinforcing adaptive coping mechanisms. It is important to acknowledge the potential impact of online recruitment methods used during COVID-19, as the shift to remote data collection may have introduced a bias in our sample. Adolescents who are more comfortable using technology or willing to discuss emotions online may be overrepresented, which could influence the coping strategies observed.

While this study provides valuable insights, limitations include difficulties in accessing hospitals for in-person data collection during the COVID-19 pandemic, which led to the use of online recruitment methods. Additionally, as this is a cross-sectional study utilizing questionnaires from multiple institutions, it would be beneficial to address potential selection bias and the constraints on the generalizability of the findings. Future research should consider family support and mental health history, as these may influence coping strategies and overall adjustment.

In conclusion, the results confirm that coping strategies evolve significantly during the cancer treatment process. Adolescents tend to rely more on strategies like worry, avoidance, and self-blame during treatment, reflecting the stress and uncertainty of this phase. As treatment progresses, these strategies decrease, particularly self-blame, indicating an adaptation to the emotional demands of the illness. Additionally, gender differences in coping strategies highlight the importance of tailored interventions to address the unique emotional needs of both males and females.

## Figures and Tables

**Table 1:** Mean differences in the different coping strategies according to the period of illness.

Strategies Coping	t	gl	Average Treatment Process	Average Follow-Up Process	Difference in Averages	*p*	Partial Eta Squared
As	0.230	199	74.98	74.45	0.527	0.819	0.000
Rp	0.110	199	76.49	76.29	0.201	0.913	0.000
Es	−0.913	199	73.38	75.17	−1.793	0.362	0.004
Pr	2.098	199	74.31	69.15	5.158	0.037	0.022
Ai	−0.069	199	68.00	68.18	−0.180	0.945	0.000
Pe	1.601	199	72.09	69.51	2.575	0.111	0.013
Hi	1.271	199	57.60	56.00	1.60	0.205	0.008
Na	0.886	199	36.18	35.06	1.115	0.376	0.004
Rt	1.860	199	28.18	25.98	2.196	0.064	0.017
So	0.131	199	54.78	54.37	0.408	0.896	0.000
Ip	2.337	199	37.56	33.33	4.222	0.020	0.027
Cu	2.929	199	31.83	26.53	5.302	0.004	0.041
Re	0.280	199	40.67	40.09	0.577	0.780	0.000
Ae	−0.145	199	37.89	38.11	−0.219	0.885	0.000
Po	−0.548	199	69.61	70.77	−1.155	0.585	0.002
Ap	1.017	199	76.72	73.29	3.434	0.310	0.005
Dr	0.530	199	82.37	81.29	1.078	0.596	0.001
Fi	0.938	199	68.76	65.59	3.170	0.349	0.004

As: seek social support; Rp: focus on solving the problem; Es: strive and succeed; Pr: worry; Ai: invest in close friends; Pe: seek belonging; Hi: wishful thinking; Na: lack of coping; Rt: reduce stress; So: social action; Ip: ignoring the problem; Cu: self-blame; Re: keeping it to oneself; Ae: seeking spiritual support; Po: focusing on the positive; Ap: seeking professional support; Dr: seeking relaxing diversions; and Fi: physical distractions.

**Table 2:** ANCOVA between age, sex, and period of illness for the coping strategy “worrying”.

Variables	Type III Sum of Square	gl	F	*p*	Partial Eta Squared
Corrected model	6721.508 ^a^	4	6.059	<0.001	0.110
Intersection	14,713.217	1	53.053	<0.001	0.213
Age	0.021	1	0.000	0.993	0.000
Sex	4494.544	1	16.207	<0.001	0.076
Period of illness	1578.822	1	5.693	0.018	0.028
Sex*Sickness period	384.343	1	1.386	0.241	0.007
Error	54,356.462	196			
Total	1,087,568.000	201			
Total corrected	61,077.970	200			

^a^: R-squared = 0.110 (Adjusted R-squared = 0.092).

**Table 3:** ANCOVA between age, sex, and period of illness fr the coping strategy “stress reduction”.

Variables	Type III Sum of Square	gl	F	*p*	Partial Eta Squared
Corrected model	800.671 ^a^	4	2.967	0.021	0.057
Intersection	1340.377	1	19.866	<0.001	0.092
Age	87.100	1	1.291	0.257	0.007
Sex	326.080	1	4.833	0.029	0.024
Period of illness	185.897	1	2.755	0.099	0.014
Sex*Sickness period	98.269	1	1.456	0.229	0.007
Error	13,224.085	196			
Total	160,176.000	201			
Total corrected	14,024.756	200			

^a^: R-squared = 0.057 (Adjusted R-squared = 0.038).

**Table 4:** ANCOVA between age, sex, and period of illness for the coping strategy “ignoring the problem”.

Variables	Type III Sum of Square	gl	F	*p*	Partial Eta Squared
Corrected model	6246.275 ^a^	4	11.370	<0.001	0.188
Intersection	4123.221	1	30.022	<0.001	0.133
Age	3.766	1	0.027	0.869	0.000
Sex	5074.668	1	36.950	<0.001	0.159
Period of illness	936.761	1	6.821	0.010	0.034
Sex*Sickness period	94.712	1	0.690	0.407	0.004
Error	26,918.650	196			
Total	282,550.000	201			
Total corrected	33,164.925	200			

^a^: R-squared = 0.188 (Adjusted R-squared = 0.172).

**Table 5:** ANCOVA between age, sex and period of illness for the coping strategy “self-blame”.

Variables	Type III Sum of Square	gl	F	*p*	Partial Eta Squared
Corrected model	1456.595 ^a^	4	2.206	0.070	0.043
Intersection	1941.785	1	11.764	0.001	0.057
Age	36.127	1	0.219	0.640	0.001
Sex	33.508	1	0.203	0.653	0.001
Period of illness	1341.351	1	8.126	0.005	0.040
Sex*Sickness period	0.863	1	0.005	0.942	0.000
Error	32,352.609	196			
Total	201,750.000	201			
Total corrected	33,809.204	200			

^a^: R-squared = 0.043 (Adjusted R-squared = 0.024).

## Data Availability

Data are contained within the article.

## References

[1] Chacón-Muñoz M.D., Cisneros-Castolo M. (2011). Modelo de intervención AFASINCA para familiares de niños con cáncer. Rev. Enfermería Inst. Mex. Seguro Soc..

[2] Yélamos C., García G., Fernández B., Pascual C., García-Saenz J.A. (2011). Medin, El Cáncer en los Niños. España: Asociación Española Contra el Cáncer. https://www.aecc.es/sites/default/files/migration/actualidad/publicaciones/documentos/guiareducida.pdf.

[3] Kornfeld D.S. (2002). Consultation-Liaison Psychiatry: Contributions to Medical Practice. Am. J. Psychiatry.

[4] Sharp K., Tillery R., Long A., Wang F., Pan H., Phipps S. (2022). Trajectories of resilience and posttraumatic stress in childhood cancer: Consistency of child and parent outcomes. Health Psychol..

[5] Wurttemberger O.R. (2016). Information and Childhood Cancer. Colomb. Med..

[6] (2022). AECC Asociación Española Contra el Cáncer, Dimensiones del Cáncer. https://observatorio.contraelcancer.es/explora/dimensiones-del-cancer.

[7] Thabrew H., Stasiak K., Hetrick S.E., Wong S., Huss J.H., Merry S.N. (2017). eHealth interventions for anxiety and depression in children and adolescents with long-term physical conditions. Cochrane Database Syst. Rev..

[8] Phelps C., Minou M., Baker A., Hughes C., French H., Hawkins W., Leeuwenberg A., Crabtree R., Hutchings P.B. (2017). Necessary but not sufficient? Engaging young people in the development of an avatar-based online intervention designed to provide psychosocial support to young people affected by their own or a family member’s cancer diagnosis. Health Expect..

[9] Puello-Alcocer E.C., Herrera-Espitia K.K., German-Orozco S.C. (2020). Sentimientos y cambios en niños y adolescentes con cáncer en Montería (2019). Rev. Cienc. Cuid..

[10] Dieluweit U., Debatin K.-M., Grabow D., Kaatsch P., Peter R., Seitz D.C.M., Goldbeck L. (2010). Social outcomes of long-term survivors of adolescent cancer. Psychooncology.

[11] Stam H., Hartman E.E., Deurloo J.A., Groothoff J., Grootenhuis M.A. (2006). Young Adult Patients with a History of Pediatric Disease: Impact on Course of Life and Transition into Adulthood. J. Adolesc. Health.

[12] van Dijk E.M., van Dulmen-den Broeder E., Kaspers G.J.L., van Dam E.W.C.M., Braam K.I., Huisman J. (2008). Psychosexual functioning of childhood cancer survivors. Psychooncology.

[13] Havighurst R.J. (1972). Developmental Tasks and Education.

[14] Lie N.K., Larsen T.M.B., Hauken M.A. (2018). Coping with changes and uncertainty: A qualitative study of young adult cancer patients’ challenges and coping strategies during treatment. Eur. J. Cancer Care.

[15] Sa D.J., Ortigosa-Quiles J.M., Riquelme-Marin A., Marti R.S. (2024). Self-Esteem and Coping Strategies in Adolescent Cancer Patients during the Period of Illness and Follow-Up. Eur. J. Investig. Health Psychol. Educ..

[16] Hensler M.A., Katz E.R., Wiener L., Berkow R., Madan-Swain A. (2013). Benefit Finding in Fathers of Childhood Cancer Survivors. J. Pediatr. Oncol. Nurs..

[17] Erickson S.J., Steiner H. (2001). Trauma and Personality Correlates in Long Term Pediatric Cancer Survivors. Child Psychiatry Hum. Dev..

[18] Hullmann S.E., Fedele D.A., Molzon E.S., Mayes S., Mullins L.L. (2014). Posttraumatic growth and hope in parents of children with cancer. J. Psychosoc. Oncol..

[19] Barakat L.P., Alderfer M.A., Kazak A.E. (2006). Posttraumatic Growth in Adolescent Survivors of Cancer and Their Mothers and Fathers. J. Pediatr. Psychol..

[20] Turner-Sack A.M., Menna R., Setchell S.R. (2012). Posttraumatic Growth, Coping Strategies, and Psychological Distress in Adolescent Survivors of Cancer. J. Pediatr. Oncol. Nurs..

[21] López J., Ortiz G., Noriega C. (2019). Posttraumatic growth in parents of children and adolescents with cancer. An. Sist. Sanit. Navar..

[22] Zebrack B.J., Chesler M.A. (2002). Quality of life in childhood cancer survivors. Psychooncology.

[23] Rosenberg A.R., Yi-Frazier J.P., Wharton C., Gordon K., Jones B. (2014). Contributors and Inhibitors of Resilience Among Adolescents and Young Adults with Cancer. J. Adolesc. Young-Adult Oncol..

[24] Gibson F., Hibbins S., Grew T., Morgan S., Pearce S., Stark D., Fern L.A. (2016). How young people describe the impact of living with and beyond a cancer diagnosis: Feasibility of using social media as a research method. Psychooncology.

[25] García A.A.G., Gómez-Maqueo M.E.L. (2016). Estilo de afrontamiento y calidad de vida en adolescentes con cáncer. Gac. Mex. Oncol..

[26] Belpame N., Kars M.C., Deslypere E., Rober P., Van Hecke A., Verhaeghe S. (2021). Coping strategies of adolescent and young adult survivors of childhood cancer: A qualitative study. Cancer Nurs..

[27] Shin H., Bartlett R., Gagne J. (2020). Integrative literature review on psychological distress and coping strategies among survivors of adolescent cancer. Oncol. Nurs. Forum.

[28] Cheng C., Lau H.-P.B., Chan M.-P.S. (2014). Coping flexibility and psychological adjustment to stressful life changes: A meta-analytic review. Psychol. Bull..

[29] McMahon E.M., Corcoran P., McAuliffe C., Keeley H., Perry I.J., Arensman E. (2013). Mediating Effects of Coping Style on Associations Between Mental Health Factors and Self-Harm Among Adolescents. Crisis.

[30] Villa A.C., Retamal G.C. (2014). Franklin de Martínez, Vivencias y significados de adolescentes sobrevivientes al cáncer. Trilogía Cienc. Tecnol. Soc..

[31] Poch F.V., Carrasco M.G., Moreno Y.G., Cerrato S.M., Aznar F.C. (2015). Los estilos y estrategias de afrontamiento y su relación con el bienestar personal en una muestra de adolescentes. An. Psicol..

[32] Compas B.E., Jaser S.S., Bettis A.H., Watson K.H., Gruhn M.A., Dunbar J.P., Williams E., Thigpen J.C. (2017). Coping, emotion regulation, and psychopathology in childhood and adolescence: A meta-analysis and narrative review. Psychol. Bull..

[33] Bottesi G., Spoto A., Trevisson E., Zuccarello D., Vidotto G., Cassina M., Clementi M. (2020). Dysfunctional coping is related to impaired skin-related quality of life and psychological distress in patients with neurofibromatosis type 1 with major skin involvement. Br. J. Dermatol..

[34] Ramírez J.S., González G.M.C. (2019). Evaluación de síntomas en niños y adolescentes con cáncer: Revisión integrativa. Rev. Cienc. Cuid..

[35] Faul F., Erdfelder E., Buchner A., Lang A.G. (2014). G*Power.

[36] Frydenberg E., Lewis R. (1996). ACS. Escalas de Afrontamiento para Adolescentes.

